# Telephone intervention in the promotion of self-efficacy, duration and exclusivity of breastfeeding: randomized controlled trial

**DOI:** 10.1590/1518-8345.2777-3140

**Published:** 2019-04-29

**Authors:** Anne Fayma Lopes Chaves, Lorena Barbosa Ximenes, Dafne Paiva Rodrigues, Camila Teixeira Moreira Vasconcelos, Juliana Cristina dos Santos Monteiro, Mônica Oliveira Batista Oriá

**Affiliations:** 1 Centro Universitário Estácio do Ceará, Fortaleza, CE, Brasil.; 2 Universidade Federal do Ceará, Faculdade de Farmácia, Odontologia e Enfermagem, Fortaleza, CE, Brasil.; 3 Universidade Estadual do Ceará, Departamento de Enfermagem, Fortaleza, CE, Brasil.; 4 Universidade de São Paulo, Escola de Enfermagem de Ribeirão Preto, Centro Colaborador da OPAS/OMS para o Desenvolvimento da Pesquisa em Enfermagem, Ribeirão Preto, SP, Brasil.

**Keywords:** Self Efficacy, Breast Feeding, Nursing, Health Promotion, Evidence-Based Nursing, Communications Media, Autoeficácia, Aleitamento Materno, Enfermagem, Promoção da Saúde, Enfermagem Baseada em Evidências, Meios de Comunicação, Autoeficácia, Lactancia Materna, Enfermería, Promoción de la Salud, Enfermería Basada en la Evidencia, Medios de Comunicación

## Abstract

**Objective:**

to evaluate the effect of a telephone intervention on the self-efficacy of puerperal women in the duration and exclusivity of breastfeeding.

**Method:**

randomized controlled trial composed of 85 breastfeeding mothers at 2 months and 77 at 4 months. The sample was randomized into two groups, control and intervention. The intervention consisted of a telephone follow-up performed at seven, 15 and 30 days after delivery using the precepts of Motivational Interview and Self-Efficacy in Breastfeeding.

**Results:**

self-efficacy in breastfeeding at 2 months was similar in both groups (p = 0.773). However, at 4 months, the intervention group presented higher self-efficacy than the control group (p = 0.011). There was a difference between groups in the duration of breastfeeding at 2 months (p = 0.035). At 4 months, the intervention group remained in breastfeeding when compared to the control group (p = 0.109). Both groups did not show differences in exclusive breastfeeding at two (p = 0.983) and four months (p = 0.573).

**Conclusion:**

the telephone educational intervention was effective in improving self-efficacy and duration of breastfeeding, but not exclusivity. (ReBEC: UTN: U1111-1180-5341).

## Abstract (pt)

**Objetivo:**

avaliar o efeito de uma intervenção telefônica na autoeficácia de puérperas na duração e exclusividade da amamentação.

**Método:**

estudo experimental randomizado controlado envolvendo 85 lactantes aos dois meses e 77 aos quatro meses. A amostra foi randomizada em dois grupos: controle e intervenção. A intervenção consistiu em um acompanhamento telefônico realizado aos sete, 15 e 30 dias pós-parto utilizando os preceitos da Entrevista Motivacional e da Autoeficácia em Amamentar.

**Resultados:**

com dois meses a autoeficácia em amamentar foi semelhante nos dois grupos (p=0,773). Todavia, com quatro meses, o grupo intervenção apresentou maior autoeficácia que o grupo controle (p=0,011). Evidenciou-se diferença entre os grupos na duração do aleitamento materno aos dois meses (p=0,035). No quarto mês, o grupo intervenção se manteve em aleitamento materno quando comparado ao grupo controle (p=0,109). Ambos os grupos não apresentaram diferenças na exclusividade da amamentação aos dois (p=0,983) e quatro meses (p=0,573).

**Conclusão:**

a intervenção educativa por telefone foi eficaz para melhorar a autoeficácia e duração do aleitamento materno, mas não a exclusividade. (ReBEC: UTN: U1111-1180-5341).

## Abstract (es)

**Objetivo:**

evaluar el efecto de una intervención telefónica en la autoeficacia de puérperas en la duración y exclusividad de la lactancia.

**Método:**

estudio experimental aleatorizado controlado envolviendo 85 lactantes a los dos meses y 77 a los cuatro meses. La muestra fue aleatorizada en dos grupos: control e intervención. La intervención consistió en un acompañamiento telefónico realizado a los siete, 15 y 30 días post-parto utilizando los preceptos de la Entrevista Motivacional y de la Autoeficacia en Lactancia.

**Resultados:**

con dos meses la autoeficacia en amamantar fue semejante en los dos grupos (p=0,773). También, con cuatro meses el grupo intervención presentó mayor autoeficacia que el grupo control (p=0,011). Se evidenció diferencia entre los grupos en la duración de la lactancia materna a los dos meses (p=0,035). En el cuarto mes, el grupo intervención se mantuvo en lactancia materna cuando comparado al grupo control (p=0,109). Ambos grupos no presentaron diferencias en la exclusividad de la lactancia a los dos (p=0,983) y cuatro meses (p=0,573).

**Conclusión:**

la intervención educativa por teléfono fue eficaz para mejorar la autoeficacia y duración de la lactancia, pero no la exclusividad. (ReBEC: UTN: U1111-1180-5341).

## Introduction

Despite the support of national and international health agencies for breastfeeding, early weaning is still an evident aspect among Brazilian nursing mothers, being a challenge to be overcome^(^ ^1^ ^)^. Evidence indicates the increase in breastfeeding (BF) and exclusive breastfeeding (EBF) until 2006, but with relative stabilization until 2013. This demonstrates the importance of strengthening implemented actions and expanding new strategies to promote breastfeeding^(^ ^2^ ^)^.

To modify this scenario, health experts aim to propose interventions based on modifiable factors, such as a proposal capable of improving women’s behavior in relation to breastfeeding. In this context, women’s self-efficacy in breastfeeding is analyzed, which can be conceptualized as the mother’s confidence in breastfeeding her child successfully, which involves knowledge and skill. This factor has been shown to have a positive effect on the duration and exclusivity of BF, promoting in the woman the feeling that she is able to modify her behaviors aiming at better health conditions for both her and her child^(^ ^3^ ^)^.

Several technologies have been used to improve maternal self-efficacy in breastfeeding and the prevalence of BF and EBF (workshops, educational booklets, album, telephone). Among these, telephone use has been increasingly used, being seen as a useful tool capable of promoting BF, proving to be effective when the interventions are performed in the long term during the puerperium and by health experts with mastery and experience in breastfeeding^(^ ^4^ ^)^.

A US study of 298 women that used a telephone contact intervention developed by prenatal breastfeeding consultants up to six months after delivery found that women in the intervention group (IG) had longer duration of BF and were more likely to continue the EBF^(^ ^5^ ^)^. A randomized experimental study involving 461 Nigerian women, in which counseling on BF was implemented through meetings with songs and dramatizations, telephone follow-up and text messages, found that women from the IG were more likely to practice EBF with one month (Odds Ratio: 1.6, p = 0.10), 3 months (OR: 1.8, p <0.05) and 6 months (OR: 2.4, p <0.01)^(^ ^6^ ^)^.

Several aspects have already been addressed during the telephone interventions, such as breastfeeding benefits and techniques, cultural aspects, difficulties and psychological support. However, no research has specifically addressed self-efficacy in breastfeeding. Our hypothesis is that interventions aimed at this construct may generate significant repercussions for the success of breastfeeding. In this context, the relevance of the present research is based on the fact that its findings will subsidize a possibility that can be added to those already used in primary health care as a way to facilitate access, guidance, support and follow-up of the mothers and their children with regard to infant feeding. Thus, the objective was to evaluate the effect of telephone educational intervention on maternal self-efficacy, duration and exclusivity of BF.

## Method

This is a controlled Randomized Clinical Trial (CRT) conducted in the period from May to November, 2015, in a District Hospital in the city of Fortaleza, Ceará. The inclusion criteria were being in the immediate puerperium, single full-term gestation with newborns hospitalized in rooming-in (RI), being practicing BF and having at least one telephone number for contact. We excluded women whose children presented deficiencies that prevented breastfeeding, presented some type of contraindication for breastfeeding and impaired hearing. The criteria for discontinuation were maternal or newborn death during the course of the study, interruption of the BF before the intervention was completed, and failure to answer telephone calls after three attempts at different days and times.

For the sample calculation, we used a formula for comparative group studies, adopting the following values: Z5% = 1.96, z20% = 0.84, p1 = proportion of the outcome in the control of 30%, p2 = proportion of the outcome in the 55% experiment, n = sample size, confidence coefficient = 95%, test power = 80%. Thus, by replacing the values, 57 puerperal women would be required for each group. However, a safety percentage of 15% was added based on the losses of a study that addressed self-efficacy in breastfeeding using the telephone^(^ ^7^ ^)^ for possible telephone losses, making up a total of 66 puerperal women per group, totaling 132 postpartum women. Participants were allocated randomly in two groups:

- Intervention Group (GI): Telephone educational intervention. In addition to the assistance and routine individual service activities provided by the child-friendly hospital professionals, the women received an educational intervention by telephone. The intervention consisted of a telephone call lasting seven minutes, on average, made by an experienced nurse and lactation educator, in which she initially introduced herself and recalled the approach in the rooming-in, in order to establish a bond with the infant. Subsequently, using a form that followed the principles of the Motivational Interview (MI), the evoking-informing-evoking technique was used, which is recommended to change patients’ behaviors in a collaborative way, based on their motivation^(^ ^8^ ^)^. At each call, guidance was given on two items on the scale to which women showed lower self-efficacy in the rooming-in; these guidelines were based on the instrument created by the researcher, based on the Breastfeeding Self-Efficacy Scale - Short Form (BSES-SF)^(^ ^9^ ^)^ and in the Serial Album “I can breastfeed my child”^(^ ^10^ ^)^, which addressed issues on technique and interpersonal thinking on breastfeeding. The doubts of the women were solved and, when necessary, they were guided to seek the institution’s milk bank.

- Control Group (CG): Women received only the routine guidelines of the child-friendly hospital, that is, individual routine service activities.

After the initial approach to the women in the rooming-in, the randomization was performed in “blocks”, 13 blocks of 10 puerperal women and one block of two puerperal women. This type of randomization was important for equitable initial distribution between groups to facilitate logistics in collecting data from subsequent phases. Randomization occurred by a computerized algorithm performed by a first statistician. Thus, each puerperal woman was allocated to participate in a group based on chance, that is, with the same chance of being distributed in one of the comparison groups.

This research involved a team, encompassing nurses and nursing academics, previously trained to evaluate outcomes. These people were blinded as well as the statistician responsible for the analysis. However, the researcher responsible for the intervention and the research participants were not blinded.

The research was divided into three phases. The first phase took place on a day-to-day basis in the recruitment of puerperal women for a period of three months in the rooming-in unit. During the admission to the obstetric ward, the puerperal women were approached about their consent to participate in the research, after receiving explanation on the objectives and benefits thereof. Subsequently, the participants answered a form containing sociodemographic, obstetrical and breastfeeding data and the BSES-SF, which assessed the participants’ maternal self-efficacy. At the end of the study, the following primary outcomes were assessed: self-efficacy of women in breastfeeding, duration and exclusivity of breastfeeding.

The BSES-SF was validated in Brazil, with Cronbach’s alpha of 0.74, showing to be a reliable instrument^(^ ^11^ ^)^. It is composed of 14 items randomly distributed in two domains (technique and intrapersonal thoughts) related to maternal confidence in breastfeeding, which has a Likert type scale ranging from 1 to 5 points. Mothers are classified as follows: Low efficacy: 14 to 32 points; Average efficacy: 33 to 51 points; High efficacy: 52 to 70 points. This instrument is self-applied when the participant has the ability to read and answer to the questions. In view of the public involved in this research, the application was through an interview conducted by the researchers.

In the second phase, the intervention was made to the IG women through three telephone contacts within one month, in the days previously established with the patients, being at 7 days, 15 days and 30 days after childbirth. In the third phase, also by telephone, the evaluation of the primary outcomes was performed based on BSES-SF and on a specific form developed by the authors, applied to both groups.

**Figure 1:**
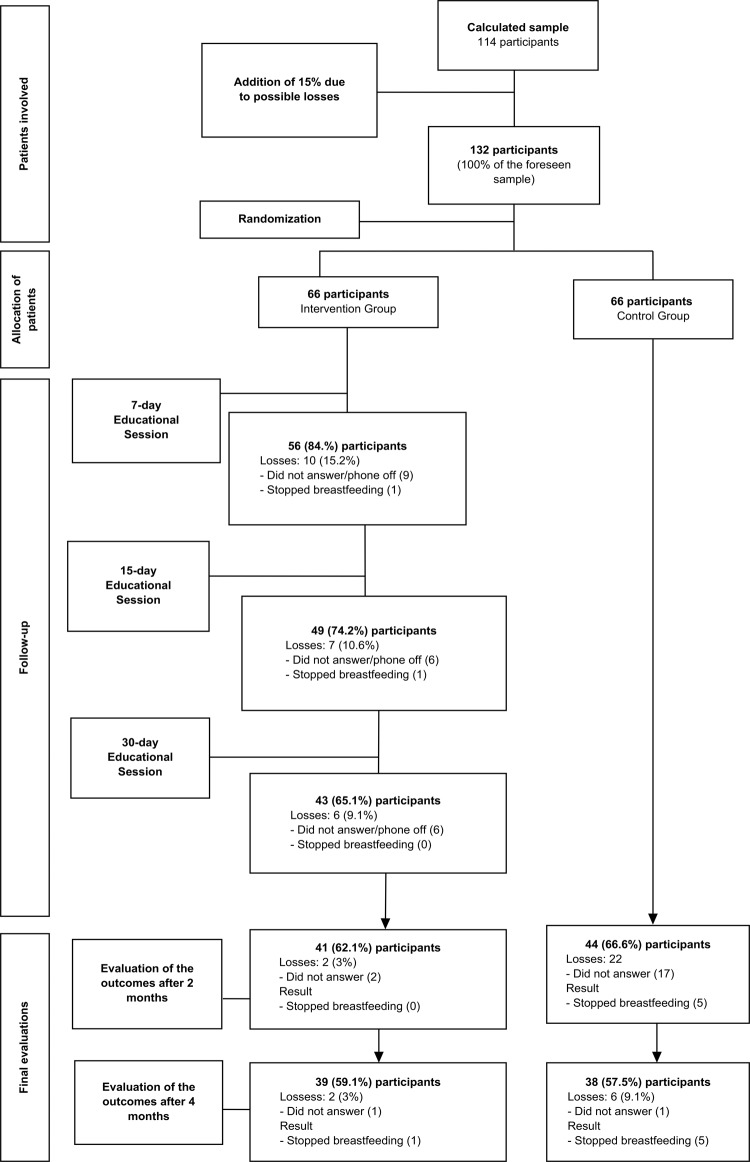
Flowchart of the phases and follow-up of participants

Of the 132 participants evaluated for eligibility, only 77 composed the final sample due to the discontinuity criteria, in which there was 40.9% of loss, as detailed in Figure 1.

The data obtained were compiled in the Statistical Package for the Social Sciences (SPSS) program, version 20.0. Continuous variables were expressed as medians with a 95% confidence interval, and categorical variables in absolute and relative frequencies. For the comparisons between the groups, we used the chi-square, Fisher, Pearson and Mann-Whitney U tests.

The study was approved by the Ethics and Research Committee of the Federal University of Ceará (Opinion 1,026,156) and registered in the Brazilian Registry of Clinical Trials (ReBEC) (UTN: U1111-1180-5341).

## Results

Comparisons of sociodemographic and obstetric variables indicate that there was no statistically significant difference between groups (Table 1). The mothers of the CG had a lower median age (CG: 22; IG: 24.5), but with similar median of years of schooling (CG: 11; IG: 11.5).

**Table 1:** Sociodemographic and obstetric data of the participants. Fortaleza, CE, Brazil, 2015

Variable	Intervention (n=66)		Control (n=66)		p-value^‡^

	Md*±SD^†^	n(%)	Md*±SD^†^	n(%)	
Age (years)	24.5 ± 7.4		22.0 ± 6.4		0.484^§^
1st quartile P25	19.5		19		
3rd quartile P75	29		29.0		
Range of schooling (years of study)	11.0 ± 3		11.5 ± 2.9		0.237^§^
1st quartile P25	08		09		
3rd quartile P75	13		13		
Family income^||^					0.712^¶^
Less than one wage		6 (9.1)		7 (10.6)	
From one to three wages		60 (90.9)		59 (89.4)	
Marital status					**0.018** ^**^
Married/Stable union		57 (86.4)		45 (68.2)	
Outros		9 (13.6)		21 (31.6)	
Occupation					0.738**
Housewife		37 (56)		30 (42.7)	
Maid		15 (22.7)		16 (24.2)	
Other		14 (21.2)		20 (30.3)	
Parity					0.541^¶^
Primipara		34 (51.5)		40(60.6)	
Multipara		32 (48.5)		26(39.4)	
Previous breastfeeding practice					0.080**
Yes		31 (96.9)		21 (80.8)	
No		1 (3.1)		5 (19.2)	

*Median; †Standard deviation; ‡*p-value*; §Mann-Whitney test; || Minimum wage: R$ 788.00, 2015, Brazil; ¶Chi-square test; **Fisher’s exact test

Mothers of the GI had a higher prevalence of married/stable marital status (CG: 68.2%; IG: 86.4%), as well as of housewife as occupation (CG: 42.7%; IG: 56%). These differences were not significant, except for marital status, since women living in married/stable unions had better levels of self-efficacy in breastfeeding.

There was no difference between the groups in relation to the obstetric history, although the CG presented a majority of primiparous women and the lowest percentage of mothers with previous breastfeeding experience.

**Figure 2:**
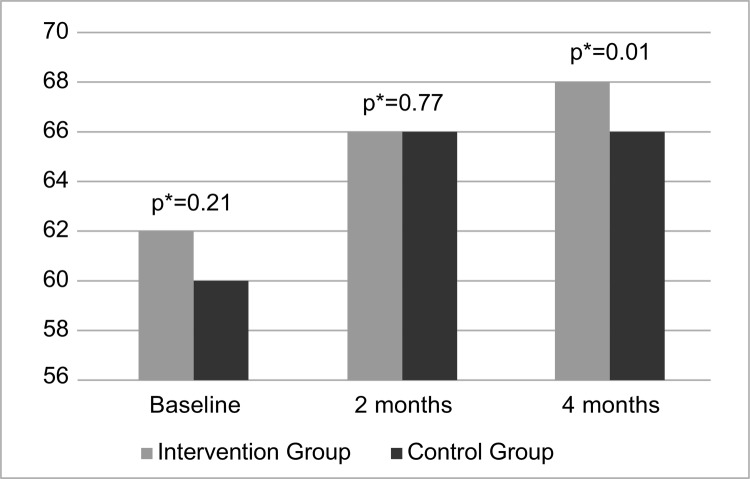
Intergroup comparison of median self-efficacy scores in breastfeeding over time. Fortaleza, CE, Brazil, 2015

Figure 2 shows the median self-efficacy scores in both groups over time.

*Mann-Whitney U Test

The analysis showed that the median self-efficacy scores were the same in the short term (two months). However, in the long term (four months), it was evidenced that the IG obtained higher levels of self-efficacy when compared to the CG. This leads us to conclude that the telephone intervention has increased women’s self-efficacy in breastfeeding in the medium term.

**Figure 3:**
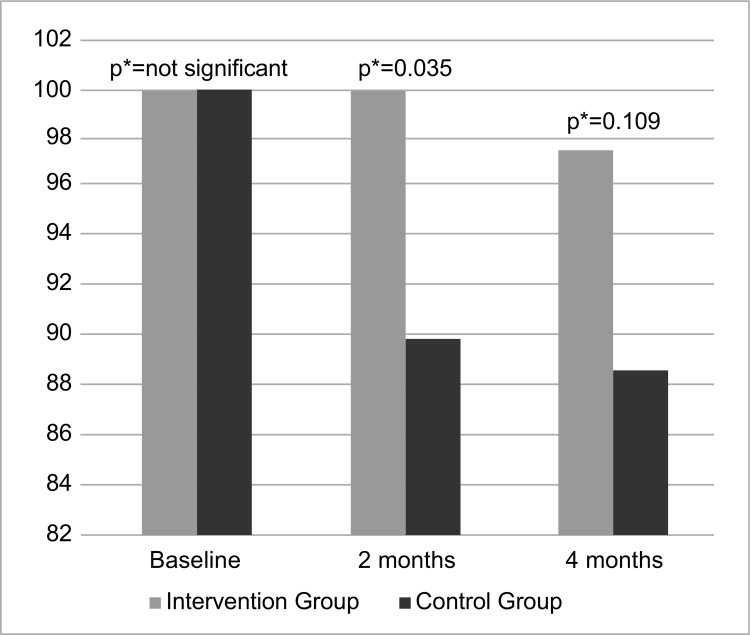
Intergroup comparison of duration of breastfeeding over time. Fortaleza, CE, Brazil, 2015

Figure 3 shows that the educational intervention was effective in maintaining BF in the short term (two months) in the IG to 100% of the women who remained in the study until two months; while the CG presented decrease. With regard to the fourth month, most women in the IG remained in BF when compared to the CG, but it was not statistically significant.

*Mann-Whitney U Test

**Figure 4:**
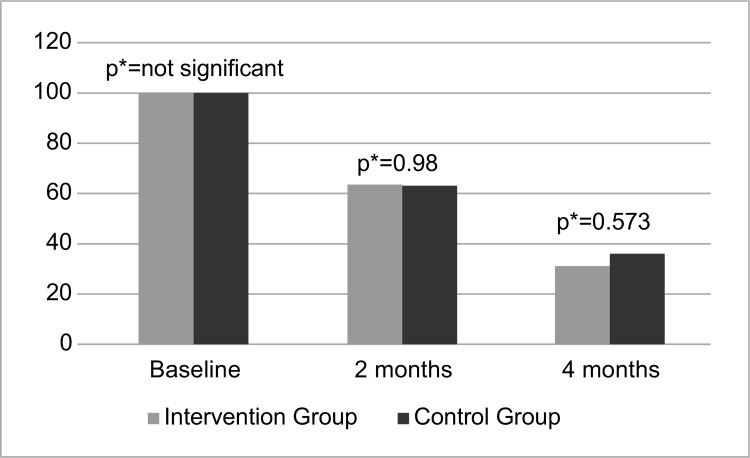
Intergroup comparison of exclusive breastfeeding over time. Fortaleza, CE, Brazil, 2015

The intergroup comparison of the exclusivity of BF indicates that both groups (CG/IG) presented minimal differences regarding the exclusivity of BF at two and four months (Figure 4). Thus, it is evident that the educational intervention did not influence the exclusivity of BF.

*Pearson’s Chi-Square

## Discussion

The analysis of the sociodemographic variables revealed that age did not influence the self-efficacy of breastfeeding, although some research have shown that the younger the age, the shorter the length of breastfeeding. This fact justifies that women be constantly stimulated through group actions directed at pregnant and puerperal women to enable them and support them so that they can breastfeed up to six months^(^ ^12^ ^)^.

On the other hand, marital status influenced maternal self-efficacy in breastfeeding. Women living with their partner may have increased self-efficacy in breastfeeding, since partner support may be a protective factor in confidence to breastfeeding, making it critical to adherence to breastfeeding^(^ ^13^ ^-^ ^15^ ^)^. Thus, the nurse is of utmost importance to guarantee qualified attention to this specific public since prenatal care in order to achieve a positive repercussion in the beginning and duration of BF.

Among the women of the IG, there was a predominance of occupation as housewives; however, this difference between the groups did not significantly influence breastfeeding. Nevertheless, the literature points out that this aspect may favor exclusive breastfeeding, considering that women who work out of home feel more distressed with the child’s adaptation to a new food pattern and offer the bottle prematurely^(^ ^16^ ^)^.

Despite the greater predominance of primiparous women in the CG, no difference was identified in the groups regarding maternal self-efficacy in breastfeeding. Primiparity is, in most cases, identified as a risk factor for low self-efficacy in breastfeeding, which may have repercussions on adherence to this practice^(^ ^6^ ^,^ ^16^ ^)^. Thus, these mothers need to receive care from the beginning of prenatal care through counseling, educational activities and a practical approach aiming at a better performance in their first experience in breastfeeding.

The educational intervention did not influence breastfeeding at two months (p = 0.773). However, it was effective at four months, increasing the self-efficacy of breastfeeding among mothers of the IG. Similar results were found in a pilot study conducted in Canada, which developed an intervention focused on self-efficacy in breastfeeding from telephone contacts. There was no difference between the groups in the self-efficacy until the second month; however, the mothers belonging to the IG presented higher levels of self-efficacy in breastfeeding at four and eight weeks after delivery compared to mothers of the CG^(^ ^17^ ^)^.

Thus, in the short term, mothers tend to maintain high self-efficacy in breastfeeding regardless of intervention. This may be related to pre-existing factors, such as guidelines received during prenatal care and previous breastfeeding experience. In view of this, such strategies are essential to sustain mothers’ confidence for a longer period and, consequently, to maintain BF and EBF^(^ ^18^ ^)^.

The educational intervention was able to maintain BF at two and four months, showing to be effective in maintaining BF in both the short and long term. Regarding the exclusivity of BF, the intervention developed did not influence this aspect, regardless of the time. A recent research has pointed out that there are several factors that contribute to discontinuation of exclusive breastfeeding (low milk production, difficulty in attachment, breast complications and lack of confidence in breastfeeding)^(^ ^13^ ^)^. Thus, in order to overcome this complex challenge, health professionals need to expand their area of intervention so that interventions address different problems.

In the face of globalization, nursing has been using Information and Communication Technologies (ICTs) as a way to develop care in the different health settings, and the telephone is an effective tool for communication^(^ ^19^ ^)^. In the present study, the intervention performed by telephone was developed during four weeks through the guidance of a trained nurse. Comparing the findings of this study with an American study in which a telephone intervention was carried out by lactation consultants certified by the International Board of Lactation Consultant Examiners (IBLCE) for up to 72 hours after delivery, one can identify better BF and EBF rates in the Brazilian study. The duration of breastfeeding was 4.3 weeks shorter in the IG than in the CG (p = 0.08), which was also observed at 30 and 90 days (p = 0.10 and p = 0.08, respectively). The duration of EBF was 4.7 weeks shorter in the IG than in the CG^(^ ^20^ ^)^.

Although the previously presented studies show significant limitations, such as specific public and small sample size, the findings allow us to understand that educational interventions carried out for a short period show gaps in their efficacy^(^ ^4^ ^)^.

The only study found in the literature conducted for a short period that obtained a satisfactory result in the duration and exclusivity of BF had as intervention a telephone follow-up aimed at the specific difficulties of the mothers, being developed by nurses that were lactation consultants up to four weeks after delivery. Mother of the IG were more likely to maintain BF at either one month (OR: 1.63) or at two months (OR: 1.48). The intervention provided a higher rate of mothers in EBF in the IG at one month (OR = 1.89, p = 0.003)^(^ ^21^ ^)^. The factors that may have contributed to these positive findings were the fact that professionals were certified as lactation consultants and the very characteristic of the intervention, that is, being focused on the problems and doubts of the puerperal women.

On the other hand, surveys that had representative samples and whose interventions were developed for a long period had a significant effect on BF^(^ ^22^ ^-^ ^24^ ^)^, evidencing the importance of postpartum follow-up, a period seen as critical in relation to breast problems and difficulties, which favors the woman to wean prematurely.

Thus, these results show that an educational intervention carried out by means of short-term telephone support developed by trained nurses, focused on self-efficacy in breastfeeding and based on the approach of MI increases the mothers’ self-efficacy in breastfeeding and increases the duration of BF, but does not influence the exclusivity of BF.

The most relevant contribution of this research is to make evident that telephone support consists of a viable technology in the promotion of BF, especially if it is used as an educational component, and that can be idealized and applied in the health services with the aim of improving the rates of BF and EBF.

However, it is important to point out that the telephone should be considered as a form of support in the assistance to mother and child to promote BF, and should not replace direct contact, attention and care of professionals to this binomial.

The high sample loss rate (40.9%) is a limitation of this study, which restricts the generalization of the effects. However, this RCT is a pioneering initiative in Brazil that may not only fill this gap in the literature but also increase the knowledge of limitations that can be adjusted in future replications.

## Conclusion

The results of this study provide positive evidence on the effectiveness of professional telephone support for the promotion of BF. This short-term educational intervention was able to increase self-efficacy and duration of BF, but did not influence exclusivity. It is believed that this research can contribute to the innovation of the care methodology, considering that it is a new possibility of strategy to be added to those already used in the health services. However, further research is needed to explore and identify the reasons for persistently low EBF rates and to test new interventions that seek to improve these rates.
